# Drug safety and immunogenicity of tumour necrosis factor inhibitors: the story so far

**DOI:** 10.1093/rheumatology/kex434

**Published:** 2018-01-08

**Authors:** Meghna Jani, William G Dixon, Hector Chinoy

**Affiliations:** 1 Arthritis Research UK Centre for Epidemiology, The University of Manchester, Manchester, UK; 2 Centre for Musculoskeletal Research, Manchester Academic Health Science Centre, The University of Manchester, Manchester, UK; 3 NIHR Manchester Musculoskeletal Biomedical Research Centre, Manchester University Hospitals NHS Foundation Trust, Manchester Academic Health Science Centre, Manchester, UK; 4 Health eResearch Centre, Farr Institute for Health Informatics Research, University of Manchester, Manchester, UK

**Keywords:** immunogenicity, safety, pharmacoepidemiology, tumour necrosis factor inhibitors, infusion reactions, drug-induced lupus, vasculitis, thrombotic events, biomarkers, biosimilars

## Abstract

TNF-α inhibitor (TNFi) therapies have transformed the treatment of several rheumatic musculoskeletal diseases. However, the majority of TNFi’s are immunogenic and consequent anti-drug antibodies formation can impact on both treatment efficacy and safety. Several controversies exist in the area of immunogenicity of TNFis and drug safety. While anti-drug antibodies to TNFis have been described in association with infusion reactions; serious adverse events (AEs) such as thromboembolic events, lupus-like syndrome, paradoxical AEs, for example, vasculitis-like events and other autoimmune manifestations have also been reported. The expansion of the biologic armamentarium, new treatment strategies such as introduction/switching to biosimilars and cost-saving approaches such as TNFi tapering, may all have a potential impact on immunogenicity and clinical sequelae. In this review we evaluate how evolution of biologics relates to drug safety and immunogenicity, appraise relevant evidence from trials, spontaneous pharmacovigilance and observational studies and outline the areas of uncertainty that still exist.

**Rheumatology key messages**

- Differences in biologic structure may lead to differential immunogenicity rates and subsequent adverse events.
- Risk of infusion-reactions, lupus and vasculitis-like events may be mitigated by addition of concomitant DMARDs.
- Treatment stratification using therapeutic drug monitoring to predict safety and effectiveness may be possible.

## Introduction

Biologic agents such as TNF-α inhibitors (TNFi’s) have significantly improved outcomes in rheumatological conditions, including RA, PsA and AS. However, up to 40% of patients do not respond to TNFis from the outset or lose response over time, the latter often being attributed to immunogenicity of the drug. Immunogenicity refers to the ability of a biotherapeutic to induce an immune response, leading to anti-drug antibodies (ADAbs), with its potential effects on pharmacokinetics and bioavailability. TNFi drug levels and ADAbs are promising biomarkers of treatment response in rheumatic diseases [1–5], although impact on drug safety is less well understood and is often misinterpreted in the literature.

Immunogenicity of TNFi’s has been linked to serious adverse events (AEs) including infusion/allergic reactions, thrombotic events, autoimmune reactions [such as drug-induced lupus (DIL)] and paradoxical AEs, for example, vasculitis. However, often the study design and data source make conclusions about causal associations between ADAbs and AEs challenging. Repeated drug exposure can result in a break in immune tolerance to self-antigens, leading to allergic reactions, immune complex disease and subsequent AEs [6]. In drug safety assessment, immunogenicity testing is now mandatory prior to approval of new biopharmaceuticals by both the US Food and Drug Administration (FDA) [7] and the European Medicines Agency (EMA) [8]. Nevertheless, historic examples of life-threatening consequences of immunogenicity to other biopharmaceuticals have been well described, often emerging later in the drug life-cycle. The discussion around immunogenicity has recently intensified with the introduction of biosimilars, concerns about switching from generics, the continual discovery of novel drug targets and subsequent biotherapeutics (with immunogenic potential). In this review, we therefore examine the association between immunogenicity and drug safety, specifically in the context of rheumatic diseases, as well as describing future strategies in TNFi management, where such understanding is relevant.

## Historic significance of immunogenicity and drug safety

**Fig. 1:**
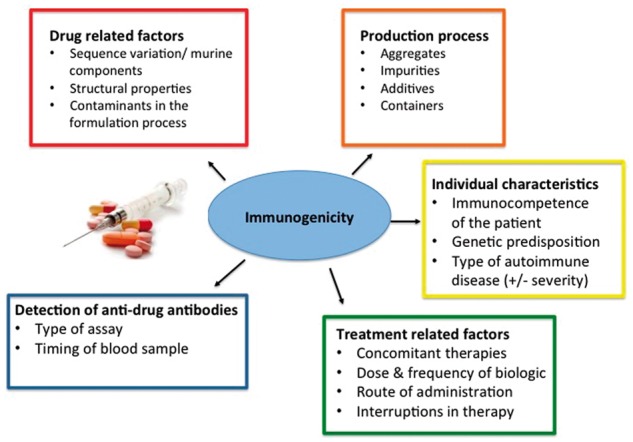
Factors affecting immunogenicity of biotherapeutics This figure illustrates the main factors that may influence immunogenicity of a biologic at different stages in the drug’s life-cycle. Multiple sources of protein aggregation exist at various points such as product manufacture, storage, shipping and drug infusion. In addition, containers of the product may lead to exposure to foreign particles such as rubber or silicone particles from stoppers and may act as immunological adjuvants. Post manufacture and drug approval, treatment-related factors and the detection of anti-drug antibodies (green and blue boxes) are potential modifiable factors on immunogenicity from a clinician perspective.

We now know several factors may affect immunogenicity (Fig. 1); however, it was initially attributed to a protein’s foreign composition. Immunological complications of animal insulin therapy for diabetes mellitus became evident almost a century ago. IgG-insulin antibodies lead to immune-mediated insulin resistance, lipoatrophy and serious allergic reactions such as serum sickness [9]. With the breakthrough of recombinant DNA technology, came the introduction of human homologues (such as hormones, growth factors and interferons), produced on the basis of sequences identical to those found in human genes. However, these were also found to induce ADAbs [10], with consequent altered pharmacokinetics, efficacy and reported immunogenic reactions, including lupus-like events (LLEs) and cutaneous vasculitis [11, 12].

One of the more alarming instances of immunogenicity leading to life-threatening safety concerns is pure red cell aplasia (PRCA) in chronic renal failure patients treated with recombinant human erythropoietin (EPO). EPO-induced antibodies neutralize all exogenous recombinant EPO and cross-react with endogenous EPO, leading to severe sudden onset anaemia. Despite widespread use of recombinant human EPO, this complication remained undiscovered until a published case series in 2002 linking PRCA with anti-EPO antibodies [13], prompting larger-scale investigations by FDA and European regulators. It was later discovered this occurred due to relatively minor drug formulation changes, following replacement of human serum albumin with glycine and polysorbate 80 in 1998.

Aspects within the manufacturing process can result in altered properties that may significantly affect patient safety [14]. These include minor structural changes in sequence variation and glycosylation to use of concomitant therapies [15] (Fig. 1). A major consideration leading to confusion regarding immunogenicity rates for biologics is the assay type used for detection (Fig. 1). ELISAs, frequently used in trials and some observational studies, often underestimate ADAbs in circulation in the presence of free drug compared with more drug-tolerant assays [16]. The FDA and EMA outline recommendations for risk mitigation in relation to such factors in their respective guidelines [7, 8].

## Evolution of biologics and differences in TNFi structure

The first mAb OKT3, licensed in 1986 by the FDA, had significant problems due to immunogenicity. It was a fully murine immunoglobulin targeting the CD3 receptor to reduce post-transplant rejection [17]. However, OKT3 induced strong human anti-murine antibody responses when administered to patients, resulting in reduced effectiveness and anaphylactic reactions. Initial improvements in mAb technology led to development of chimeric proteins, with murine variable but human constant regions and a more favourable clinical risk–benefit profile. Infliximab is a chimeric antibody containing 25% mouse-derived amino acids and 75% human-derived amino acids; consequently, human anti-chimeric antibodies can ensue.

To reduce the proportion of murine sequences, further engineering of mAb led to humanization, the grafting of murine regions for human sequences, resulting in a significantly less immunogenic product (although complementarity-determining regions of variable regions remain of murine origin). Certolizumab pegol is a humanized protein containing mouse-derived amino acid sequences in the complementarity-determining regions. A third approach was the development of fully human antibodies (e.g. adalimumab, golimumab). Fully human and humanized antibodies should carry a lower risk for inducing immune responses in humans than murine or chimeric antibodies. However, adalimumab and golimumab still have the potential to induce marked immune responses.

Infliximab, adalimumab and golimumab are full-length, bivalent IgG mAbs, whereas certolizumab pegol is a monovalent fragment antigen-binding 1 (Fab1) antibody fragment covalently linked to polyethylene glycol, but devoid of the crystalline fragment (Fc) portion of IgG. Etanercept differs from other TNFi biologics, as it is a fully human, recombinant dimeric fusion protein consisting of the Fc fragment of human IgG1 fused to two extracellular binding portions of human TNFR2/p75. The fusion/hinge portion of the molecule may contain new epitopes that could be recognized as foreign by the immune system, thus leading to ADAb formation. Biosimilars are protein equivalents of generic biologic drugs that arrive onto the market after patent expiration of an originator therapeutic protein [18]. The complexities in the manufacturing of such biotherapeutics mean that even subtle and undetectable differences from their originator products may lead to unexpected alterations in their biological function and immunogenicity, and potential changes in their safety and efficacy [19].

## Immune-mediated adverse events

**Fig. 2:**
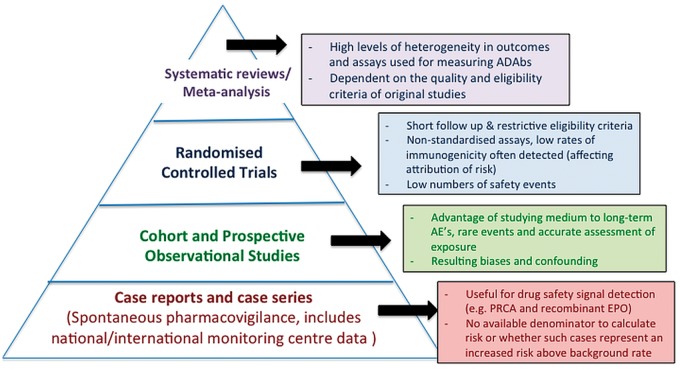
Hierarchy of evidence and assessment of drug safety in association with immunogenicity Spontaneous pharmacovigilance data includes adverse event monitoring by the FDA and MHRA. While randomized controlled trials are the gold standard in the assessment of efficacy, prospective observational cohort studies provide the most useful information in the assessment of risk of reported safety events in association with immunogenicity. Each study design has its benefits and outlined limitations in the assessment of drug safety. AEs, adverse events; EPO, erythropoietin; PRCA, pure red cell aplasia.

Immunogenicity of TNFi agents resulting in adverse drug reactions is well documented. Classic phenomena mediated by immune complexes [including serum sickness, bronchospasm and Arthus reactions (local vasculitis, with deposition of immune complex and complement activation)] have been described in RA and IBD [20]. However, several uncertainties exist due to (i) issues with traditional study designs (Fig. 2), (ii) lack of well-phenotyped patients assessed with reliable assays and (iii) lack of long-term data. Recent concerns include risk of AEs such as infusion reactions (due to formation of ADAbs) with newer intravenous biosimilars, relevance of autoantibody seroconversion (secondary to immunogenicity, with risk of autoimmune reactions such as LLE), paradoxical reactions [e.g. vasculitis-like events (VLEs)] and thromboembolic events. We discuss these areas next in relation to published evidence. Randomized control trials (RCTs) and spontaneous pharmacovigilance data are referred to, especially if there are few data (Fig. 2); however, where possible safety evidence from observational studies are discussed.

## Infusion/hypersensitivity reactions

### Infliximab and biosimilar infliximab

Infusion reactions are the most frequently reported in ADAb-positive infliximab-treated patients, ranging between 4 and 15% [21–26]. Other related AEs have also been observed, including urticarial rashes [21, 27], exanthema [21] and Quincke’s oedema [25], and rarely infliximab-induced allergic dyspnoea and Guillain-Barré syndrome in small observational studies [25, 27]. ADAbs to infliximab are mainly of the IgG isotype, also associated with neutralizing potential. Since infliximab-related severe reactions (chest tightness, hypotension, respiratory distress) can resemble type I allergic reactions, the role of IgE-ADAbs has been considered [28]. However the majority of infusion reactions do not appear to be IgE-ADAb mediated [29]. Use of concomitant MTX lowers immunogenicity rates and subsequent infusion reactions, as observed as early as the initial trials [30].

While observational data from biosimilar agents is currently lacking, infusion-related reactions in association with ADAb formation have been reported in RCTs. In the PLANETRA trial, comparing CT-P13 and originator infliximab in RA, detection of immunogenicity was comparable by 30 weeks (48.4% *vs* 48.2%, respectively) [31]. Infusion-related reactions occurred in 20 (6.6%) CT-P13 patients, of which 9 were ADAb positive, and in 26 (8.3%) patients on originator infliximab, of whom 18 were ADAb positive. In the PLANETAS trial that compared the same drugs in AS [32], of the 11 (9.1%) and 13 (11%) patients who developed ADAbs by 30 weeks in the CT-P13 and infliximab groups, respectively, infusion reactions were observed in 1 (3.1%) and 3 (11.1%) patients. Therefore, trials thus far seem to demonstrate a comparable rate of ADAbs with biosimilar and originator infliximab, but with some initial differences in infusion reactions reported.

### Association of auto-antibodies and immunogenicity

All TNFi agents have been associated with asymptomatic immunological alterations to autoimmune pathology with systemic manifestations. The development of ANA positivity has been reported in 31–63% of infliximab-treated patients, in 16–51% of adalimumab-treated patients and in 12–48% of etanercept-treated patients, within prospectively observed RA cohorts [33, 34]. However, the significance of seroconversion in the absence of clinical manifestations has been questioned.

Recent observational data has suggested that a proportion of patients on mAb-based TNFi agents may develop ANA and dsDNA antibodies due to immunogenicity, which may act as a surrogate marker of impending treatment failure—as seen in two small studies in psoriasis [35, 36]. ANA/dsDNA seroconversion rates have also been observed at higher rates in TNFi-treated secondary non-response patients in RA [34, 37] and psoriasis patients [35], with a direct association with ADAbs seen in infliximab-treated patients [36]. Similarly in IBD patients, pANCA positivity may predict lower clinical response in mAb-treated patients [38]. Feasibly, patients predisposed to developing immunogenicity, may also be prone to seroconversion of other antibodies, for example, ANA, dsDNA and ANCA. At the extreme end of this autoantibody spectrum, clinical evolution to LLE and VLE appears less frequent.

### Lupus-like events

While TNFi safety has been studied extensively over the last 17 years with acceptable safety and tolerability profiles, rare autoimmune-mediated phenomena have more recently emerged as a concern. Within the sub-group of autoimmune-driven AEs, LLE and VLE appear to be the most common, with the vast majority of cases occurring in RA [39]. The association of TNFi’s with DIL or symptoms within the lupus spectrum has been well reported; however, the association directly with immunogenicity has been less clear. LLE may present with classical dermatological signs, low complement levels and an increased frequency of anti-dsDNA antibody titres, but unlike in typical DIL, the incidence of anti-histone antibodies is low [40].

### RCTs and spontaneous pharmacovigilance

Induction of autoantibodies and LLE was observed initially in the earliest clinical trials of infliximab [41, 42]. Similarly, one case of LLE was observed in the PREMIER study (adalimumab monotherapy arm) [43] and RAPID2 study in certolizumab-treated RA patients [44]. While, the majority of early TNFi trials reported no cases of LLE [45–49], ANA and dsDNA seroconversion was commonly reported.

Most evidence about LLE characteristics comes from spontaneous pharmacovigilance. Following RCT reports of ‘infliximab-induced lupus,’ concerns were raised about etanercept in the year 2000 following publication of a case series of four LLE events [50]. Further case series have been published by de Bandt *et al.* (*n* = 12) [51], Ramos-Casals *et al.* (*n* = 92) [39] and Costa *et al.* [52] in RA and PsA. The latest LLE and VLE figures were obtained from the Medicines and Healthcare products Regulatory Agency (MHRA) drug analysis prints, and are as shown in Table 1. It is worth noting that the earliest reported event for both certolizumab and golimumab was almost a decade later than the other three TNFi agents reflecting their licensing dates, which may explain the low event numbers in Table 1.

**Table 1:** Lupus and vasculitis-like events on TNFi agents reported to the UK regulatory agency

Drug (licensing date UK for RA)	Earliest reported event to MHRA		Reported LLE cases (*n*)	Reported vasculitis cases (*n*)
Infliximab (2002)	23 July 1999		Lupus-like syndrome (63) SLE (32) Cutaneous lupus (3)	Vasculitis (29) ANCA-+ve vasculitis (2) Cerebral vasculitis (2) Vasculitic rash (8) Granulomatosis with Polyangiitis (1) Bechet’s syndrome (1) DM (1)
Etanercept (2002)	30 September 1999		Lupus-like syndrome (7) SLE (15) Cutaneous lupus (13) Lupus vasculitis (2)	Vasculitis (21) Necrotising vasculitis (2) Granulomatosis with Polyangiitis (1) Bechet’s syndrome (1)
Adalimumab (2007)	24 March 2000		Lupus-like syndrome (25) SLE (25) Cutaneous lupus (11)	Vasculitis (24) Cutaneous vasculitis (11) Necrotising vasculitis (2) Gastrointestinal vasculitis (2) Cerebral vasculitis (2) Granulomatosis with Polyangiitis (1) Bechet’s syndrome (5)
Certolizumab (2010)	25 February 2010		Lupus-like syndrome (3) Cutaneous lupus (1)	Vasculitis (1) Skin vasculitis (1) Panniculitis (1)
Golimumab (2011)	3 December 2010		Lupus-like syndrome (3) SLE (6)	Vasculitis (3) Skin vasculitis (1)

Figures from Drug Analysis Prints 31/5/2017 report, MHRA (includes biosimilars as not listed separately). The Drug Analysis Prints give a complete listing of all UK spontaneous suspected adverse drug reactions reported through the Yellow Card Scheme to the MHRA and the Government’s independent scientific committee on medicines safety, the Commission on Human Medicines. There is a high-level grouping by System Organ Class (the highest level in Medical Dictionary for Regulatory Activities) that groups together reactions that affect similar systems/organs in the body, followed by a more detailed breakdown, as outlined above. LLE, lupus-like events; MHRA, Medicines and Healthcare Products Regulatory Agency.

Interpretation of risk using spontaneous pharmacosurveillence poses a number of challenges. One of the few studies attempting to quantify drug-specific risk of LLE between mAb and etanercept used French pharmacovigilance data [53]. A disproportionality analysis was used and reporting odds ratios (RORs) were calculated. RORs are based on the rationale that in the absence of an association between the reaction of interest (LLE) and the treatment (TNFi), the ratio of LLEs observed with TNFi to the total number of reactions observed with TNFi should be the same as the ratio of LLEs observed with all other drugs to the total reactions observed with all other drugs [54]. The authors concluded that there was a significant association between TNFi agents and LLE (ROR = 7.72, 95% CI: 5.50, 10.83). The highest ROR was for infliximab and adalimumab, with almost half for etanercept, although CIs overlapped and were wide. However, first, the analysis included patients on TNFi’s for all indications. There are likely to be differences between the baseline risk of diseases (for instance RA is likely to have a high baseline risk as SLE overlap is recognized), limiting the external validity of the results. Second, patients with known SLE overlap were not systematically excluded; therefore, the risk of incident events attributed to TNFi is difficult to ascertain. Third, any notoriety bias (increased reporting of adverse drug reactions following an index case/safety alert) during the immediate post–TNFi-licensing years may significantly impact on signal detection in spontaneous reporting systems. Therefore, while useful for potential safety signals, it does not replace estimates derived from prospective observational studies.

### Observational studies and registries

Registry data assessing risk of LLE are scarce, but allow more robust estimates of absolute risk in real-world settings. The British Society for Rheumatology Biologics Register for RA (BSRBR-RA) evaluated risk of first LLE in TNF-treated RA patients compared with in TNFi-naïve patients on non-biologic DMARDs (nbDMARDs) [55]. The incidence of first LLE was low at 10/10 000 person-years in the TNFi-treated cohort (95% CI: 8, 13) (Table 2). Infliximab conferred the highest unadjusted hazard ratio (HR); however, the CI overlapped between TNFis post adjustment [55]. Following adjustment of baseline differences such as DAS28 and HAQ, the overall HR fell to 1.86 (95% CI: 0.52, 6.58) for TNFis. Risk factors for LLE included being female, non-white ethnicity and minocycline use, all of which have been associated with the risk of SLE itself [56]. Therefore, in the majority of patients, LLE may be attributed to the patient characteristics of those who receive the TNFi’s (some who may have a pre-existing predisposition to SLE, even if this was not clinically evident pre-treatment), rather than to the drug itself, but the risk remains small.

**Table 2:** Studies estimating risk of lupus and vasculitis-like events in TNFi-treated patients

References	Type of study	Diseases evaluated	Outcome studied	TNFi agent	Events on TNFi (*n*)	Median time to event (months)	Controls	Estimation of risk
De Bandt *et al.* [58]	Case series	RA	LLE	INF, ETA	22	INF (9), ETA (4)	None	Denominator based on unpublished company reports INF 15/7700, 0.19%, ETA 7/3800, 0.18%
Flendrie *et al.* [59]	Prospective cohort study	RA	All cutaneous events including LLE/VLE	INF, ADAL, ETA	LLE (1), VLE (5)	For all cutaneous events (9) For vasculitis-cutaneous events (12)	RA patients not on biologics	OR of a dermatology referral was calculated in TNFi users *vs* non-users OR = 2.26 (95% CI: 1.46, 3.50) LLE/VLE risk not calculated
Lee *et al.* [60]	Observational clinical study (single centre)	RA, AS, PsA	All cutaneous events including LLE/VLE	INF, ADAL, ETA	LLE (0) VLE (1)	Not specified	None	One patient developed leucocytoclastic vasculitis
Grönhagen *et al.* [61]	Swedish population case control study (SCLE only)	All	SCLE	Not specified (all)	4	2 months	Swedish general population	OR cases: controls 8.0 (95% CI: 1.6, 37.2)
Takase *et al.* [34]	Observational single-centre UK-based study	RA	LLE/VLE	INF, ADAL, ETA	LLE (3) VLE (2)	LLE (26) VLE (mean, 21.7)	None	Not formally assessed. 3/454 patients on first TNFi developed LLE (0.7%), 2/454 VLE (0.4%)
Moulis *et al.* [53]	French pharmacovigilance study	RA, AS, PsA, IBD	LLE	INF, ADAL, ETA	39	11	Postive control isoniazid, negative control paracetamol	Association of TNFi and lupus: ROR 7.72 (95% CI: 5.50, 10.83) using disproportionality analysis
Jani *et al.* [55]	Prospective observational study	RA	LLE/VLE	INF, ADAL, ETA, CERT	LLE (54) VLE (81)	LLE (14) VLE (12)	nbDMARD-treated cohort	LLE crude incidence rate: 10/10,000 patient-years in TNFi cohort Adjusted HR for LLE in TNFi-treated cohort compared with nbDMARD: 1.86 (95% CI: 0.52, 6.58) VLE crude incidence rate: 15/ 10 000 patient-years in TNFi cohort Adjusted HR for VLE in TNFi-treated cohort compared with nbDMARD: 1.27 (95% CI: 0.40, 4.04)

ADAL, adalimumab; CERT, certolizumab pegol; ETA, etanercept; HR, hazard ratio; LLE, lupus-like event; nbDMARD, non-biologic DMARD; OR, odds ratio; ROR, reporting odds ratio; VLE, vasculitis-like events.

Interestingly, high DAS28 and HAQ scores were also associated with higher rates of the event, while use of nbDMARDS was associated with lower rates [55], similar to the development of immunogenicity [1, 4, 5]. A limitation of the study was that patients did not have measurements of ANA pre-treatment or ADAbs during treatment to establish a potential association. A trend was observed in patients who subsequently switched to another TNFi, to have recurrent LLEs compared with switching to rituximab. It is not clear whether such observations may be immunogenicity-mediated; however, switchers with anti-infliximab antibodies may be more likely to develop ADAbs against adalimumab [57]. Switching within class (i.e. another TNFi mAb) may not be preferable either in the context of secondary inefficacy or development of autoimmune AEs, if alternative options are available. Thus a causal link between ADAbs and LLE remains under review, and it does not explain the LLEs reported in etanercept-treated patients, in whom immunogenicity is low. Studies endeavouring to quantify the risk of LLE (often with a different primary outcome) are summarized in Table 2.

### Paradoxical adverse events: vasculitis-like events

While TNFs have been used as effective treatments in severe vasculitis, they have been also reported as a cause of drug-induced vasculitis [62], and hence termed ‘paradoxical.’ Patients on TNFi agents have been reported to develop a spectrum of vasculitis-like events (VLEs), ranging from cutaneous to life-threatening systemic manifestations, including large-vessel vasculitis [63, 64] and Granulomatosis with Polyangiitis [65, 66]. Serology can be consistent with lupus and/or ANCA positivity/aPLs [58, 66, 67]. Complement activation may be induced by immune complexes (in reaction to the drug) that deposit on small vessels [68]. The cytokine imbalance secondary to TNF-α inhibition may also play a role, with a shift from a Th1 to a Th2 profile (upregulating antibody production) [68, 69].

### RCTs and spontaneous pharmacovigilance

Initial RCTs for the five TNFi’s did not reveal a signal of concern regarding VLEs [30, 48, 49, 70, 71]. Ramos-Casals and colleagues [39] reported on behalf of the Spanish Society of Internal Medicine Study Group on Autoimmune Diseases (including the cases reported and quarterly MEDLINE searches of autoimmune manifestations while on TNFi’s). Cutaneous leucocytoclastic vasculitis was the most common manifestation, reported in 79 cases, with cases also of cutaneous necrotizing vasculitis (*n* = 8), peripheral neuropathy (*n* = 6), cutaneous lymphocytic vasculitis (*n* = 4), Henoch–Schönlein purpura (*n* = 2), APS (*n* = 2), polyarteritis nodosa, temporal arteritis, urticarial vasculitis (*n* = 1 for each) and unspecified vasculitis (*n* = 6). Similarly a French national retrospective survey [69] revealed 39 VLE cases (21 in etanercept, 15 in infliximab, 2 in adalimumab and 1 in an investigational TNFi drug), with no denominator of the total exposed patients to calculate an accurate incidence.

### Observational studies

Few studies have evaluated the risk of vasculitic manifestations on TNFi agents (summarized in Table 2 [59–61]). From a single-centre RA cohort, 454 patients on biologic agents were evaluated to assess whether serial ANA monitoring provided any additional clinical benefit over a median follow-up of 4.8 years [34]. The two VLEs described occurred in seropositive patients and developed cutaneous involvement requiring change in treatment (one ANA positive, one without). Although ADAbs were not formally measured, there was an association between secondary inefficacy and ANA seroconversion in this study.

Data from the BSRBR-RA suggests the risk of both systemic and cutaneous VLE is low in TNFi-treated RA patients, with a crude incidence of 15/10 000 person-years (95% CI: 12, 19), the risk highest being in the first year of TNFi treatment [55]. Factors associated with higher rates of VLE were seropositivity, high disease duration, baseline DAS28 and HAQ scores—all associated with rheumatoid vasculitis. Following adjustment, the VLE risk for TNFi- compared with for nbDMARD-treated patients was not significant (HR = 1.27; 95% CI: 0.40, 4.04), supporting the likelihood that the majority of VLEs may indeed be rheumatoid vasculitis driven by disease activity, rather than associated with TNFi’s (as adjusting for confounding by indication using propensity scores alters the HR to non-significant). In rare cases, systemic features, including renal vasculitis and cANCA PR-positive vasculitis with life-threatening manifestations, were reported, with three deaths in the TNFi group. Factors associated with lower rates of VLEs included again being on concomitant nbDMARDs (MTX, SSZ), were also associated with lower immunogenicity [15, 72]. Causality and a clear association is difficult to ascertain between ADAbs and VLE due to a lack of well-characterized studies and the rarity of such events. It can be difficult to distinguish between rheumatoid vasculitis and TNFi-induced reactions, as the former is associated with high disease activity, which in turn is the indication for TNFi.

## Thromboembolic events

### RCTs, spontaneous pharmacovigilance and observational studies

RCTs have not signalled a safety concern in relation to venous thromboemboli (VTE) in exposed TNFi patients [30, 48, 49, 70, 71]. Case series with TNFi’s such as etanercept have reported a possible association with VTE [73]. In a study evaluating 85 spontaneous reports of thromboembolic events on TNFi’s, 23 had their autoantibodies measured, of whom 13 had either ANAs and/or aPLs [74]. It may be plausible that patients who seroconvert to ANA-positive status are more likely to develop ADAbs; the combination of TNF-inhibition and the predisposition of some patients to LLE, including APS, leads to an increase in reported thromboemboli [75].

One of the most concerning reports linking adalimumab-induced ADAbs and thromboemboli was by Korswagen *et al.* [76] in 2011, who reported a higher incidence of VTE, serious arterial thromboembolic events and vasculitis-type phenomena. While highlighting a potential safety signal, this association has not been replicated and there were issues with the measurement of risk. First, the initial three cases prompted a retrospective review of all cases in ADAb-phenotyped patients to specifically look for thromboembolic events, inadvertently introducing reporting bias in a study not established to measure safety. Second, there may be an issue of misclassification, because events included myocardial infarction, strokes, transient ischaemic attacks (TIAs), peripheral arterial thrombosis, and digital ischaemia, as well as superficial vein thrombosis, deep vein thrombosis and pulmonary emboli. However, from subsequent literature there may be differential effects of TNFi’s on the risk of arterial thromboemboli and VTE. Myocardial infarction risk may be reduced due to beneficial effects on inflammation [77], while VTE risk may be increased initially [78] or unchanged [79]. Third, we know from published work using the same cohort [1], that there were baseline differences in disease severity between patients with and without ADAbs: these were not adjusted for and therefore introduce confounding by indication. RA patients with high disease severity may simply be more likely to develop thromboemboli. Indeed, there does appear to be an increased risk of VTE in RA patients compared with in the general population, regardless of their ADAb status [80].

Two large prospective observational studies to date investigating the question of thromboembolic risk in TNFi-treated patients compared with nbDMARD patients did not find an associated overall increased risk [78, 79], although stratification by ADAb status was not possible. The former study did report an increased risk of VTE over the first 180 days in all biologics, not just TNFi-agents [78], suggesting the effect may be related to disease activity (but this could not measured in the US claims database). Therefore, at present an increased thromboembolic risk does not appear to be a concern in the majority of TNFi-exposed or reported ADAb-positive patients; however, whether there is a strata of patients who may benefit from closer monitoring is not clear.

### Future strategies in TNFi-treated patients

The landscape of biologic treatment is rapidly evolving. The introduction of biosimilars has led to another paradigm shift in the use of biologics, with potential wider access of high-cost drugs and significant impact on health-care budgets. Strategies for minimizing costs to health-care systems, such as tapering, are regularly being tested and advocated in patients in remission [81]. Such changes, however, may have an impact on immunogenicity, which is discussed next.

### Tapering, immunogenicity and drug safety

Interruption in therapy or dose reduction has been associated with an increase in immunogenicity (Fig. 1). In one of the early dose-tapering studies, van den Bembt *et al*. [82] tested whether infliximab dose in 18 RA patients treated with 5 mg/kg could be reduced to 3 mg/kg. Detectable ADAbs were found in two and four patients, respectively, before and after dose decrease. Most patients successfully reduced their dose, with the exception of two patients. One developed a persistent flare, which subsided following increasing the infliximab dose, while the other developed LLE that required drug cessation. More recently, several RCTs have tested a variety of TNFi tapering strategies: while the primary outcome has been efficacy, to date there have not been any safety signals suggesting increased AEs in the tapering subgroup [83–85]. However, due to the rarity of immune-mediated AEs, the small numbers of patients in RCTs and the lack of well-characterized observational studies, an association of higher rates of ADAbs and a subsequent increase in immune-mediated AEs is difficult to quantify.

### Switching to biosimilars

Currently, one of the main concerns of switching from originator products to a biosimilar is immunogenicity and drug safety, and this has led to stricter FDA and EMA regulations regarding interchangeability. Long-term extension of original RCTs or national trials such as NOR-SWITCH, evaluating switching, have not highlighted potential serious safety concerns; however, again these studies are limited by low numbers of events and patients [86, 87]. In the NOR-SWITCH study, infusion reaction rates were comparable between the originator and biosimilar infliximab (4% *vs* 2%), and one patient on biosimilar infliximab developed a femoral arterial thrombus (their ADAb status was not specifically reported).

A national Danish study described 1-year outcomes of 802 infliximab-treated patients who switched to a biosimilar [88]. While ADAbs were not measured, in RA, biosimilar retention rate was poorer following switching, with monotherapy associated with lower retention. Of the 16% who stopped treatment, 28% (*n* = 37) stopped due to AEs, including skin rashes, infusion/allergic reactions, dyspnoea and fever/flu-like illness (all relevant to immunogenicity). Recently, the first case report of serum sickness post switching from infliximab to inflectra in RA has also been reported [89]. The rarity of such AEs requires on-going vigilance via adequately designed registries in order to obtain conclusive exclusion of any meaningful risk.

### Stratification of benefit and risk

While switching between TNFi’s and biologics occurs commonly (either within class or to non-TNFi therapy), currently there is conflicting evidence regarding the best subsequent strategies following treatment failure or development of AEs on a population level [57, 90]. Stratification based on measurement of immunogenicity and drug levels, has been proposed on the basis of observational studies [2, 4, 91, 92], and early pharmacological monitoring may predict which patients will benefit from an earlier shift to alternative treatment, thereby individualizing treatment approaches. In addition, patients on higher doses of TNFi drugs are more susceptible to serious infections, as evidenced by a recently published large meta-analysis across 106 trials [93]. Therefore, it may be feasible that patients with higher drug levels may be more susceptible to certain AEs such as infection. Well-designed pragmatic trials and integration of clinical, patient-reported outcome data, biomarkers and drug-safety signals within electronic patient records may offer opportunities to eventually allow better identification of the strata of patients who may have differential safety and effectiveness profiles.

## Conclusions

Structural and functional differences between TNFi agents may lead to differential consequences, including immunogenicity, disease-specific efficacy and AEs. Lessons can be learnt from historic instances of unforeseen immunogenicity and AEs following minor structural changes to the drug, with newer agents such as biosimilars. While there is clear evidence that immunogenicity is associated with infusion reactions to intravenous TNFi’s such as infliximab, there is a paucity of data to allow robust associations with longer-term effects of ADAbs on drug safety. Incidence of the most common immune-mediated AEs such as LLE and VLE appears low. Since the initial report of VTEs being associated with ADAbs, no prospective cohort studies have replicated this finding; however, stratification by immunogenicity status has not been possible. The issue of immunogenicity and drug safety is likely to receive more attention in relation to future treatment strategies and biosimilar switching/interchangeability and requires further evaluation to understand how best to utilize such tests as pharmacological biomarkers [82] in relation to both safety and effectiveness in clinical practice.
